# Fungal-derived selenium nanoparticles and their potential applications in electroless silver coatings for preventing pin-tract infections

**DOI:** 10.1093/rb/rbac013

**Published:** 2022-02-22

**Authors:** Xinjin Liang, Shuai Zhang, Geoffrey Michael Gadd, John McGrath, David W Rooney, Qi Zhao

**Affiliations:** 1 The Bryden Centre, School of Chemical and Chemistry Engineering, Queen’s University Belfast, Univeristy Road, Belfast BT7 1NN, UK; 2 Geomicrobiology Group, School of Life Sciences, University of Dundee, Dow Street, Dundee DD1 5EH, UK; 3 School of Pharmacy, Queen’s University Belfast, Lisburn Road, Belfast BT9 7BL, UK; 4 State Key Laboratory of Heavy Oil Processing, Beijing Key Laboratory of Oil and Gas Pollution Control, College of Chemical Engineering and Environment, China University of Petroleum, 18 Fuxue Road, Changping District, Beijing 102249, China; 5 School of Biological Sciences, Queen's University Belfast, Chlorine Gardens, Belfast BT9 5DL, UK; 6 School of Chemistry and Chemical Engineering, Queen’s University Belfast, Stranmillis Road, Belfast BT9 5AG, UK; 7 School of Science and Engineering, University of Dundee, Nethergate, Dundee DD1 4HN, UK

**Keywords:** infection, coating, silver, selenium, fungi

## Abstract

Pin-tract infections (PTIs) are a common complication of external fixation of fractures and current strategies for preventing PTIs have proven to be ineffective. Recent advances show that the use of anti-infection coatings with local antibacterial activity may solve this problem. Selenium has been considered as a promising anti-infection agent owing to its antibacterial and antibiofilm activities. In this study, selenium nanoparticles (SeNPs) were synthesized *via* a cost-effective fungi-mediated biorecovery approach and demonstrated excellent stability and homogeneity. To investigate their anti-infection potential, the SeNPs were doped in silver coatings through an electroless plating process and the silver–selenium (Ag–Se) coatings were tested for antibacterial and antibiofilm properties against *Staphylococcus aureus* F1557 and *Escherichia coli* WT F1693 as well as corrosion resistance in simulated body fluid. It was found that the Ag–Se coating significantly inhibited *S.aureus* growth and biofilm formation on the surface, reducing 81.2% and 59.7% of viable bacterial adhesion when compared with Ag and Ag–PTFE-coated surfaces after 3 days. The Ag–Se coating also exhibited improved corrosion resistance compared with the Ag coating, leading to a controlled release of Ag^+^, which in turn reduced the risk of cytotoxicity against hFOBs. These results suggest that the fungal-derived SeNPs may have potential in use as implant coatings to prevent PTIs.

**Figure:**
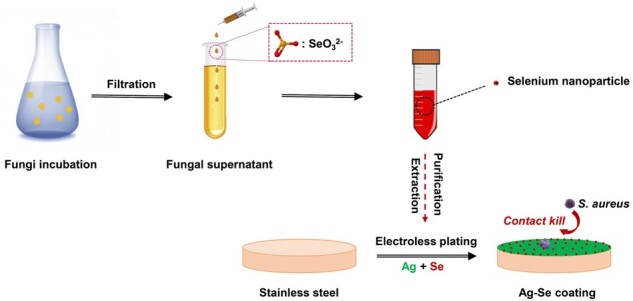

## Introduction

Pin-tract infections (PTIs) remain the most significant complication in external fracture fixation to date [1]. Numerous factors have been shown to influence the development of PTIs, and failure to resist bacterial adhesion and biofilm formation makes the pin surface a nidus for infections [2, 3]. Moreover, due to the disruption of the dermal barrier during implantation, pins can function as a conduit between the skin and underlying soft tissue and bone leading to severe complications (e.g. septic arthritis and osteomyelitis) [4]. Treatment of biofilm-related PTIs is difficult and often requires pin removal [5]. Over recent decades, concern over the risks of systematic administration of antibiotics has led a research shift to developing anti-infection coatings to inhibit/retard bacterial adhesion [6–9]. To date, only hydroxyapatite-coated pins have entered clinical use, which aims to reduce the exposure area of bone surface to bacteria while promoting new bone growth on the pin surface [10]. However, there is a lack of evidence confirming their clinical benefits in reducing PTIs [11, 12]. Recent advances in developing novel antimicrobial coatings have led to some promising candidates including nitric oxide coatings [6], photocatalyst coatings [13], hydroxyapatite-chitosan coatings [14] and chlorhexidine and iodine coatings [15]. These coatings demonstrate benefits in reducing bacterial colonization and biofilm formation although their long-term biocompatibility and technical difficulties (e.g. poor adherence of the coatings) remain to be investigated and solved [3, 5].

Silver (Ag) has long been used as an anti-infection material in medical devices (e.g. urinary catheters and wound dressings) due to its broad-spectrum antimicrobial activity. Previous studies have reported that Ag-coated pins can effectively decrease bacterial colonization and result in less infection and motion at the pin site [16, 17]. However, there are also contrary findings that the Ag coatings cannot significantly reduce bacterial adhesion when compared with the uncoated surfaces [9]. Generally, Ag releases Ag ions (Ag^+^) through an oxidative dissolution process and its antibacterial ability is Ag^+^-concentration-dependent [18, 19]. Silver nanoparticles, due to their larger surface-to-volume ratio, have a more potent antibacterial activity than bulk Ag, but the burst release of Ag^+^ (particularly during the initial period) may also induce toxicity at a cellular and organismic level [20–23]. Given the balance between antibacterial activity and toxicity, most of the Ag-coated medical devices on market have been shown to only delay early onset infections due to limited Ag^+^ release. For example, Cook *et al.* [24] reported that over 80% of the adhered bacteria on Ag coating were alive, which eventually led to the subsequent formation of a mucoid biofilm.

Selenium (Se) is a trace element in the human body and plays critical roles in maintaining thyroid hormone metabolism and DNA synthesis and protection from oxidative damage and infection [25]. Recent advances have demonstrated that selenium nanoparticles (SeNPs) can inhibit bacterial growth and biofilm formation [26–28]. Moreover, SeNPs have been reported to exhibit significantly less toxicity towards human cells than other antibacterial nanoparticles, such as Ag and copper nanoparticles [29, 30]. Despite the antibacterial mechanism remaining unclear, recent studies suggest that SeNPs may exert their antibacterial activity *via* pathways including adenosine triphosphate depletion, reactive oxygen species generation, membrane depolarization and membrane disruption [31]. Current strategies to produce SeNPs comprise traditional methods (physical and chemical methods) and biological methods. Biological methods utilizing microorganisms to generate SeNPs have cost-benefits and are environmentally friendly without producing toxic by-products or using harsh reaction conditions [32]. More importantly, biological-derived SeNPs (bSeNPs) often exhibit high stability and biocompatibility without using stabilizing or capping agents [32–34]. This feature makes bSeNPs highly suitable as coating additives. However, research has not been conducted to explore the anti-infection activity of biological-derived Se in coatings against PTIs.

In this study, we, for the first time, have developed a silver–selenium (Ag–Se) nanocomposite coating using fungal-derived SeNPs to reinforce antibacterial activity. To achieve this goal, we synthesized SeNPs with high stability *via* a microbial reduction process using supernatants from *Aureobasidium pullulans* liquid cultures, and varied amounts of SeNPs were co-deposited with Ag onto 316 L stainless steel through electroless plating. The hypothesis includes: (i) the incorporation of SeNPs could enhance the antimicrobial activity of the coatings against both planktonic and adhered bacteria; (ii) the Ag–Se coating could exhibit improved anti-corrosion property; and (iii) the Ag–Se coatings would exhibit improved biocompatibility.

## Results and discussion

### Fungal formation of SeNPs

Over the recent decades, biosynthesized nanoparticles have been regarded as a promising alternative to nanoparticles fabricated by traditional chemical or physical methods with advantages, such as improved stability, biocompatibility, bioactivity etc. [35, 36]. Compared with bacteria, fungi have proven to have higher productivity of nanoparticles due to their capability of secreting larger amounts of proteins and amino acids [37]. In this study, SeNPs were synthesized *via* a *A.pullulans*-mediated biorecovery process [33]. After 10 days, the *A.pullulans* supernatant was harvested, centrifuged and filtered, then reacted with Na_2_SeO_3_, the supernatant was discarded and the SeNPs were harvested and characterized.

**Figure 1.**
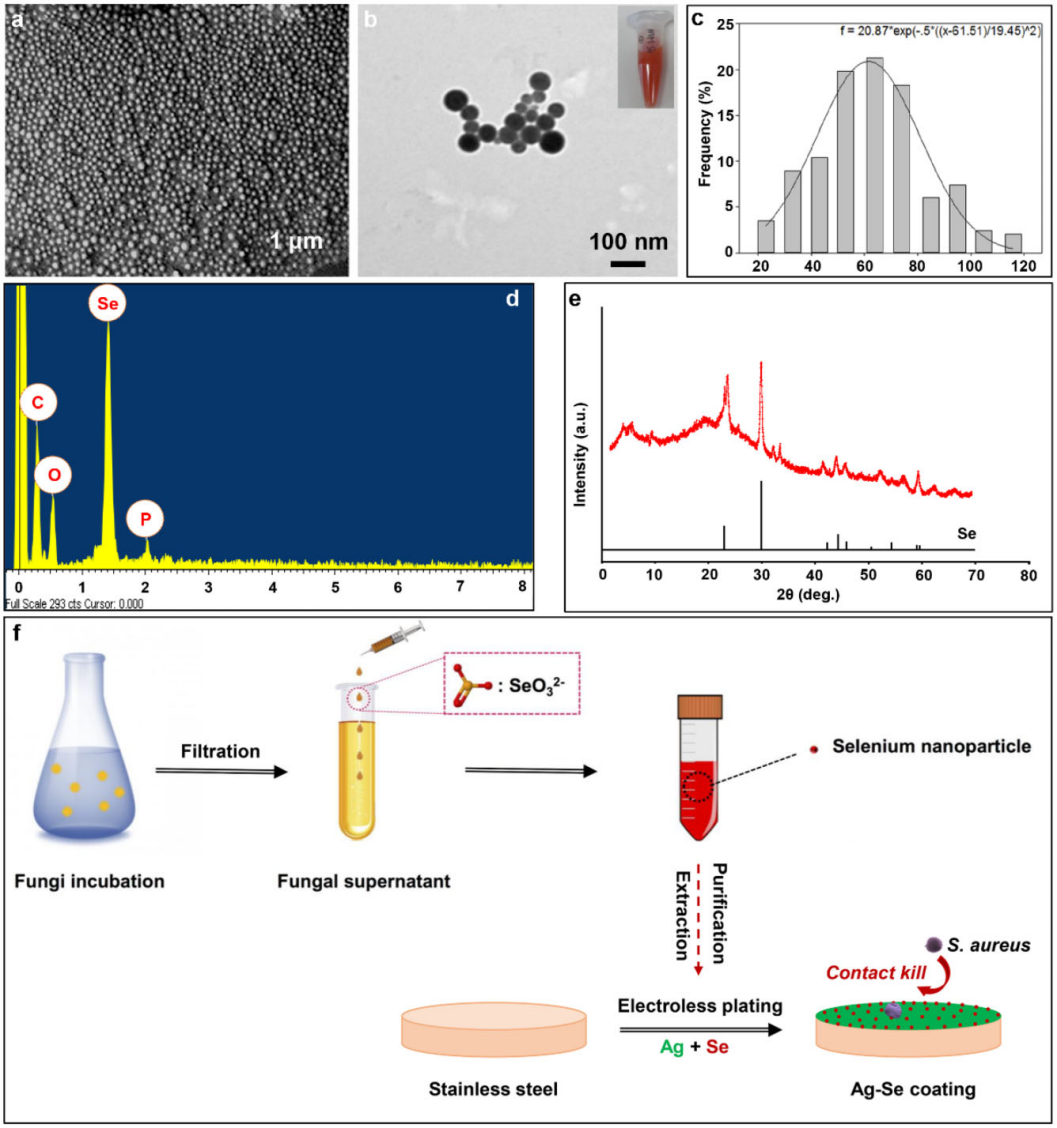
(**a**) Typical SEM image of the SeNPs harvested from *A.pullulan’s* supernatants at different magnifications; (**b**) typical TEM image of the SeNPs; (**c**) size distribution of the SeNPs and Gaussian fitting; EDX analysis (**d**) and XRD analysis (**e**) of the SeNPs; (**f**) illustrative diagrams of the fungal formation of SeNPs and the Ag–Se coating process

Technically, filamentous fungi have ability to produce SeNPs intracellularly and extracellularly. Compare to bacteria and other unicellular organisms, the fungal supernatant route makes bioprocessing and biomass handling more efficient. As shown in Fig. 1a and b, the generated particles were granular, and the average diameter ranged from 20 to 120 nm, with the highest proportion of sizes between 50 and 70 nm (Fig. 1c). Energy-dispersive X-ray spectrometry (EDX) analysis revealed the elemental composition of the particles produced in the fungal supernatant. Most particles showed peaks for carbon, oxygen, Se and phosphorus as the main elements (Fig. 1d). X-ray diffraction (XRD) data indicated that the Se-containing particles produced by *A.pullulans* showed a match to reference patterns for elemental Se. To test stability, the obtained SeNPs were stored at room temperature for 3 months and no obvious aggregation occurred. However, the mechanism of extracellular SeNPs formation by fungi remains unclear. Previous studies demonstrated that the formation of elemental Se from the reduction of selenite by fungal supernatant was the result of direct reaction with reducing organic substances, proteins and enzymes. Such reduction is a common feature of many microorganisms, which may also be attributed to some non-specific reduction by extracellular polymeric substance and proteins [38, 39].

### Preparation of Ag–Se nanocomposite coatings

**Figure 2.**
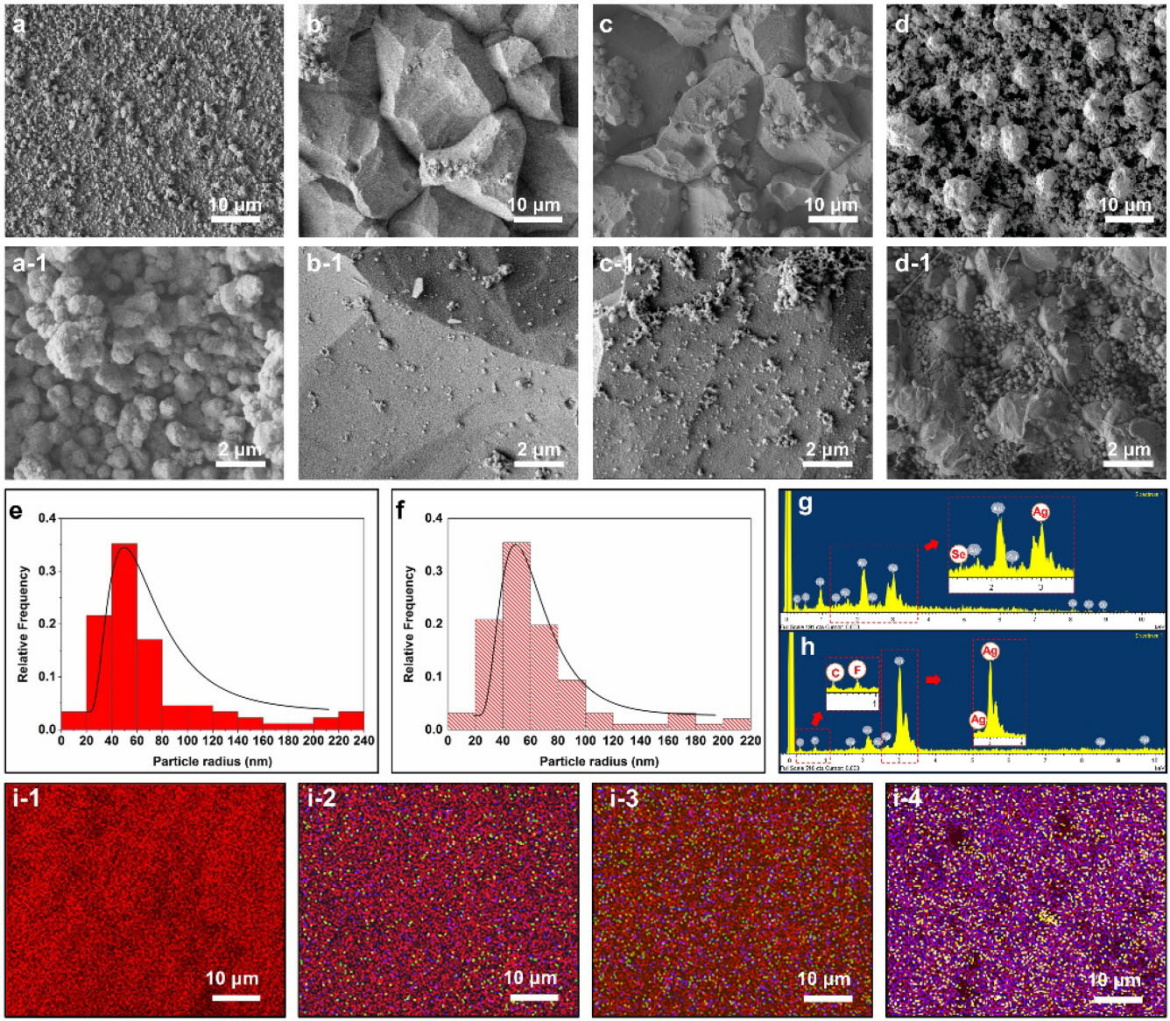
Typical SEM images of (**a**) Ag coatings; (**b**) Ag–Se (50 ml/l) coatings; (**c**) Ag–Se (100 ml/l) coatings and (**d**) Ag–PTFE coatings; (**e** and **f**) size distributions of SeNPs in Ag–Se (50 ml/l) and Ag–Se (100 ml/l) coatings; typical EDX analysis of (**g**) Ag–Se coatings and (**h**) Ag–PTFE coatings; (**i1–i4**) EDX mappings of SEM images (a)–(d) (element colour: Ag, red; C, yellow; Se, green; F, blue)

**Figure 3.**
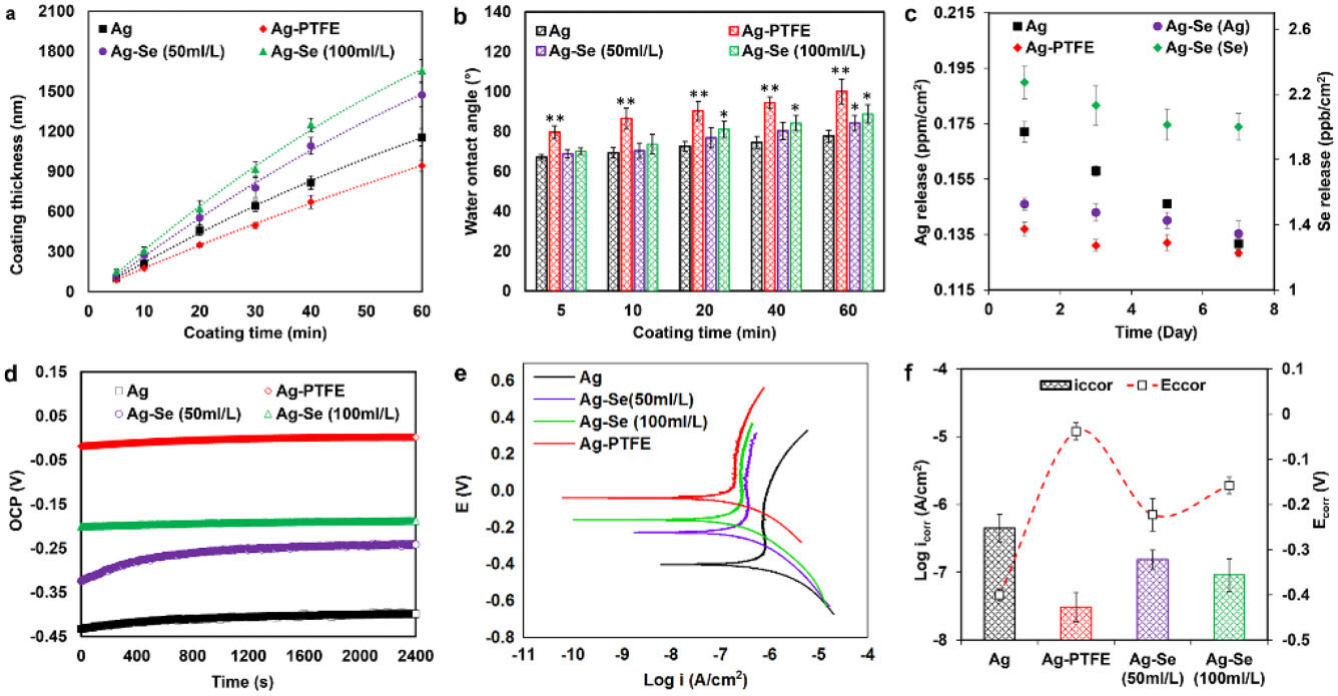
(**a**) Thickness of different coatings as a function of coating time; (**b**) WCAs for different coatings as a function of coating time; (**c**) Ag and Se (100 ml/l) release profiles over time; (**d**) open-circuit potential; (**e**) potentiodynamic polarization curves; (**f**) the *E*_corr_ and log *i*_corr_ of different coatings in SBF (*n* = 3, bars represent standard deviation of the mean; *P <* *0.05 and **P <* *0.01)

Technically, electroless Ag deposition on a pre-activated surface is a fast process and the coating solution becomes unstable over time. In order to obtain uniform Ag-based composite coatings, particles should be uniformly suspended in the plating solution during the plating process. In this study, the obtained fungal-derived SeNPs were directly mixed with electroless Ag plating solution at concentrations of 50 100 ml/l, respectively (Fig. 1f). The plating solutions remained stable without aggregation of SeNPs during the plating process. Figure. 2a–d shows the surface morphologies of Ag, Ag–Se and Ag–PTFE coatings. The Ag coating showed the smoothest surface with grain size in the range of ∼600 nm to 1.5 μm (Fig. 2a-1). After incorporation of PTFE, the surface became rougher and the size of the Ag particles became larger (∼2–5 μm). This is because the co-deposition of PTFE impedes the 3D growth of surrounding Ag islands, which in turn results in crystal accumulation of Ag in other directions. In comparison, the surface of Ag–Se coatings showed typical polygonal Ag hillocks (Fig. 2b and c). Such featured structures usually stem from breaks in the crystal lattice that generate dislocations during crystal growth. A possible reason is that the high deposition rate interrupts the equilibrium shape of Ag crystals during growth. Therefore, we measured the coating thickness over time to verify this assumption. As seen in Fig. 3a, the thickness of all types of coatings increased linearly with deposition time. The incorporation of SeNPs significantly accelerated the deposition process and a higher SeNPs concentration in the plating solution led to a faster coating deposition. Interestingly, despite all the plating solutions remaining stable during the electroless plating process, the Ag–Se coating bath decomposed the earliest followed by Ag and Ag–PTFE baths. This indicated that the SeNPs may influence the stability of the Ag-complex in the plating bath, which results in a high deposition rate. As shown in Fig. 2b-1 and c-1, along with the deposition of SeNPs, the stabilizing protein molecules were also co-deposited onto the surface. These protein molecules were negatively charged in the alkaline plating solution and may disrupt Ag-complexes, resulting in a sub-stable plating solution leading to a higher deposition rate. However, a higher concentration of SeNPs in the plating bath did not result in significant particle aggregation in coatings, suggesting good stability of SeNPs (Fig. 2e and f). EDX analysis (Fig. 2g and h) confirmed the presence of SeNPs and PTFE within the Ag matrix and their uniform distributions were further verified by EDX mapping (Fig. 2i).

The surface wettability of coatings was monitored by measuring the water contact angle (WCA) during the coating process. In general, the values of WCA of all the surfaces increased with coating time. As seen in Fig. 3b, the Ag–PTFE coatings demonstrated a significant increase in hydrophobicity, which indicated the successful co-deposition of PTFE particles in the Ag matrix. The Ag–Se coatings show higher values of WCA after 20 min deposition, which indicated that the surface became rougher due to the high deposition rate. This result is consistent with the data in Fig. 3a.

In this study, we also investigated the influence of deposition time on the bonding strength of the coatings, and the bonding strength was assessed by a standard tape test according to ASTMD 3359-02. As shown in Supplementary Fig. S1, all the Ag, Ag–PTFE and Ag–Se (10 ml/l) coatings exhibited the highest adhesion strength grade of 5B (no detachment of the squares of the lattice) within a deposition time of 1.5 h. However, the plating solution of the Ag–Se (10 ml/l) coating became unstable afterwards and the adhesion strength grade of the Ag–Se coating significantly dropped to 1B after 2 h as the coating flaked along the edges of cuts and over 50% of the area was detached (Supplementary Fig. S1c-2). In comparison, the plating solutions of Ag and Ag–PTFE coatings remained stable even after 2 h and the coatings still exhibited relatively good adhesion strength with adhesion strength grades of 4B and 3B, respectively. It should be noted that the deposition rate of the Ag–Se coating was much higher than that of the Ag and Ag–PTFE coatings, and the higher thickness (with the same deposition time) can often decrease coating adhesion due to the higher the inner structural stress. Moreover, the sub-stable plating solution of the Ag–Se coating could also lead to a decrease in the coating quality. As for the Ag–PTFE coatings, as the PTFE nanoparticles are chemically inert, an increase in deposition time results in a higher PTFE content in coatings, thus leading to a decrease in the adherence of the coating to the substrate.

### Corrosion resistance

For bone fixation pins, apart from biocompatibility, high corrosion resistance is the most important consideration [40]. Despite stainless steel being widely used as an anti-corrosion material for manufacturing bone pins, numerous studies have reported toxic reactions and chronic allergies in the host after implantation [41, 42]. The acidic body fluid upon inflammation combined with low oxygen can hasten implant corrosion by delaying formation of passive oxide films. In this study, the corrosion resistance of different Ag-based coatings was assessed *via* an electrochemical method in simulated body fluid (SBF). As shown in Fig. 3d, the corrosion potentials of Ag–PTFE and Ag–Se coatings shifted towards a nobler direction, suggesting the formation of a passive film providing an improved anodic protection. The Ag–PTFE coating had the highest open-circuit potential (OCP) value indicating the best thermodynamic stability. Compared with the Ag coating, Ag–Se coatings exhibited better corrosion resistance (Fig. 3d). A higher SeNP concentration in the plating bath resulted in a higher OCP value, which indicated that the incorporation of SeNPs improved corrosion resistance. Previous studies also demonstrated that Se as an additive can enhance the anti-corrosion property of coatings [43]. The potentiodynamic polarization curves are shown in Fig. 3e. From the Tafel plots, the corrosion potentials (*E*_corr_) of Ag–PTFE and Ag–Se-coated surfaces were higher than those of Ag-coated surfaces and the corrosion current densities (*i*_corr_) were lower. The achieved *i*_corr_ values of the Ag–PTFE coating and Ag–Se (SeNPs 100 ml/l) coatings are, respectively, 3.08 × 10^−8^ and 9.13 × 10^−8^ A/cm^2^ (SeNPs 100 ml/l), which are nearly one and a half magnitudes lower than that of the Ag coating (4.22 × 10^−7^ A/cm^2^). These results demonstrate that the incorporation of SeNPs can improve corrosion resistance. Enhanced anti-corrosion properties were further verified from the inductively coupled plasma (ICP) results (Fig. 2c) as the release of Ag^+^ from the Ag–Se-coated surface was significantly slower than that from the Ag-coated surface. The potential mechanisms for this are further discussed in the next section.

### Antibacterial efficacy and biofilm formation

Clinically, *Staphylococcus aureus* has been reported to be the most common causative agent of metal-related PTIs [8]. The rise in incidence has also been accompanied by an increase in antibiotic-resistant strains [44]. In this study, the antibacterial efficacy of Ag–Se coatings was evaluated *in vitro* against *S.aureus* F1557 and compared with Ag and Ag–PTFE coatings in terms of antibacterial activity against planktonic bacteria, anti-adhesion and antibiofilm activities.

**Figure 4.**
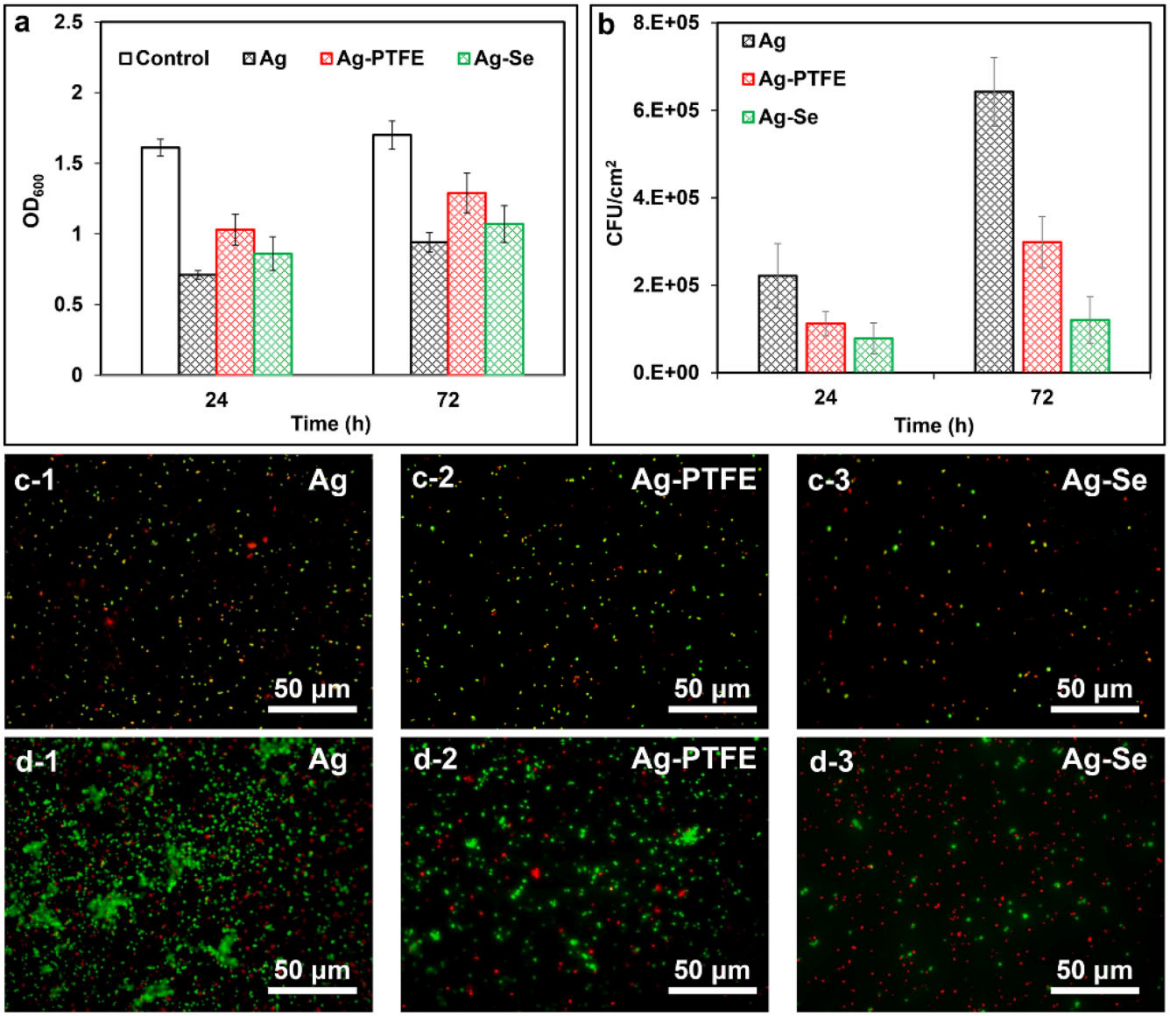
(**a**) *Staphylococcus aureus* growth in the presence of Ag, Ag–PTFE and Ag–Se (100 ml/l)-coated samples after 24 and 72 h incubation; (**b**) numbers of adhered *S.aureus* on different surfaces after 24 and 72 h incubation; typical live/dead fluorescent images of adhered *S.aureus* on different surfaces after (**c**) 24 h and (**d**) 72 h incubation (*n* = 6, bars represent standard deviation of the mean)

As seen in Fig. 4a, the Ag coating exhibited the best antibacterial activity against planktonic bacteria followed by Ag–Se (100 ml/l) and Ag–PTFE coatings. This can be ascribed to the higher release rate of Ag^+^ during the test period (Fig. 3c). In theory, the oxidation process of Ag^0^ in water is slow and only trace amounts of Ag dissolve and be released into solution. Therefore, the difference in Ag release rate can be explained in combination with Fig. 2a–d. The size of Ag particles in the Ag coating was the smallest, which enabled the most rapid dissolution of Ag^+^. Despite the Ag particle size of the Ag–PTFE coating being smaller than that of the Ag–Se coating, the enhanced surface hydrophobicity (WA∼104°) retarded its interaction with water molecules, leading to a slow Ag^+^ release. Figure 3c also shows that the Ag–Se coating was capable of releasing relatively constant trace amounts of Se (∼2 ppb) in SBF during the test period, suggesting a strong bond between SeNPs and the coating matrix. To further mimic the real condition, the release profile was also studies in the presence of serum. As seen in Supplementary Fig. S2, and the release of Ag^+^ and Se (Supplementary Fig. S2) in serum-supplement SBF was enhanced, which was in agreement with previous study [45]. However, it remains unclear that whether the released Se had antibacterial effects against planktonic bacteria. To verify this, we treated the suspensions of *S.aureus* and *Escherichiacoli* with Ag^+^ (AgNO_3_) at the same concentrations and no significant difference in optical density (OD) values was found when compared with the corresponding Ag–Se coatings. These results indicated that the release of Se from the Ag–Se coating had no antibacterial activity against *S.aureus* and *E.coli*.

The anti-adhesion activity of different coatings was compared and the bacterial density on surfaces was determined using a viable plate count method. As shown in Fig. 4b, the Ag–Se (100 ml/l) coated surfaces exhibited the best anti-adhesion efficacy throughout the test period, reducing bacterial adhesion by 64.7% and 30.3%, respectively, as compared with the Ag and Ag–PTFE-coated surfaces after 24 h. After 72 h incubation, the Ag–Se-coated surface reduced 81.2% and 59.7% of bacterial adhesion when compared with Ag and Ag–PTFE-coated surfaces, respectively. As only viable cells can be counted by this method, we further stained the adhered bacteria to investigate the anti-adhesion mechanism of Ag–Se coating. As seen in Fig. 4c, the Ag-coated surface showed the highest level of bacterial adhesion with the ratio of live/dead cells being about 1.6 while the ratios for Ag–PTFE and Ag–Se-coated surfaces were about 2.1 and 0.4, respectively. The Ag–Se-coated surface displayed the strongest bactericidal effect against adhered bacteria despite the Ag coating releasing the highest level of Ag^+^ and exhibited the strongest antibacterial activity against planktonic bacteria in the first 24 h. The results indicate that the Ag–Se coatings may possess an antibacterial contact-killing property against *S.aureus*. Moreover, after 72 h incubation, biofilm formation was observed on the Ag-coated surface and apparent bacterial aggregation was observed on the Ag–PTFE-coated surface. In comparison, biofilm formation or cell aggregation was not observed noticed on the Ag–Se-coated surface, indicating the Ag–Se effectively inhibited bacterial growth and biofilm formation. A possible mechanism contributing towards the inhibition effect is that SeNPs can catalyse the oxidation of intracellular thiols and generate singlet oxygen, which further leads to the death of bacteria [46]. Similar phenomenon was also observed with *E.coli*. As seen in Supplementary Figs S3 and S4, all the coatings demonstrated significant antibacterial activity against planktonic *E.coli* and zone of inhibition results further proved that Ag–Se coating and Ag coating were capable of inhibiting bacterial growth by releasing antibacterial components. Considering the lower level of released Ag^+^ from the Ag–Se coating, the enhanced inhibition effect could be attributed to the released Se. However, their anti-adhesion activity showed no significant difference within 72 h (Supplementary Fig. S3b). This could be due to the potent antibacterial Ag^+^ that effectively kill the planktonic *E.coli* during the test period as Gram-negative bacteria are more sensitive to Ag^+^ [18].

### Cell assays

**Figure 5.**
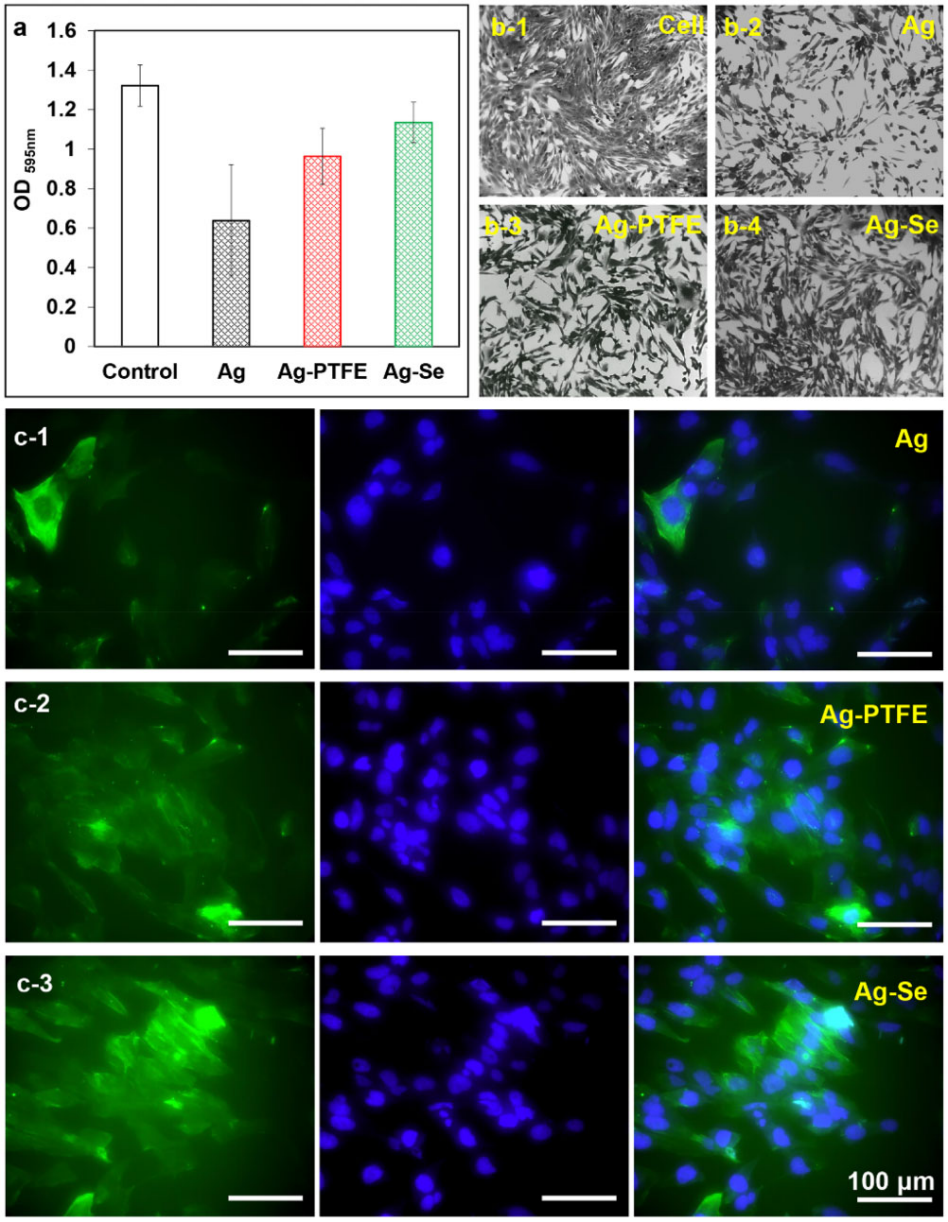
(**a**) Absorbance values of the crystal violet assay after 24 h incubation; (**b**) typical images of cells cultured with different samples after CVS; (**c**) typical confocal microscopy images of hFOBs attached on Ag, Ag–PTFE and Ag–Se surfaces after 24 h incubation (scale bar corresponds to 100 μm). Typical images are shown from one of several examinations (*n* = 6, bars represent standard deviation of the mean)

For external fracture fixation pins made of conventional metallic materials, such as stainless steel and titanium, in addition to infections, their bioinertness can often lead to delayed osteoblast response and incomplete osseointegration, which serve as a critical factor that determines the implant success. A general strategy to solve this problem is to combine implants with bioactive materials to facilitate the attachment and proliferation of osteoblasts on the implant surface so that a proper foundation of extracellular matrix can be laid down for the growth of new bone tissue, i.e. to promote the integration of the orthopaedic implant into bones. Early studies have shown that Se could promote the function of osteoblasts and speed up the formation of implant osseointegration, thereby improving the strength of the implant fixation [47]. To verify this, the adhesion of human foetal osteoblasts (hFOBs) on different coatings was studied to explore their osseointegration potentials. As seen in Fig. 5c, the cells were stained blue [4′,6-diamidino-2-phenylindole (DAPI)] for nuclei and green (Alexa Fluor 488 Phalloidin) for actin filaments. The cell numbers on the Ag-coated samples were the lowest when compared with that on Ag–PTFE and Ag–Se-coated samples. This could be ascribed to the accumulated Ag^+^ on surface that inhibited cell proliferation, which was also found in the crystal violet staining (CVS) assays. The number of cells on the Ag–PTFE and Ag–Se-coated surfaces are close and the cells exhibit a spindle shape. Interestingly, the size of the spindles on the Ag–PTFE-coated surfaces is smaller, which indicates that the cells on Ag–PTFE coatings suffer a higher stretching resistance. As shown in Fig. 5c-1, the cells on Ag-coated surfaces demonstrated a typical polygonal morphology. The difference in morphology could be due to the different surface hydrophobicity. In this study, the Ag–PTFE coating has the most hydrophobic surface, and the incorporation of SeNPs into Ag matrix also results in an increase in surface hydrophobicity (Fig. 3b). Previous studies demonstrated that hydrophobic surfaces can significantly restrict the spreading of hFOBs when compared with hydrophilic surfaces [48]. On the other hand, the Ag–Se and the Ag–PTFE also resulted in intense staining of actin filaments. Recent studies showed different surface roughness may lead to different cell responses and cells were more stressed in response to the highest surface roughness [49]. To verify this, the surface roughness (Ra) of the coatings was determined by AFM. As seen in Supplementary Table S1, for all the coatings, the surface became rougher with deposition time. The neat Ag coating has the smoothest surface and the Ag–Se coating has the roughest surface. Figure 5c-3 shows the hFOBs attached on the Ag–Se coating with a deposition time of 1.5 h, and the surface roughness was much higher than that of Ag and Ag–PTFE coatings. We then investigated the influence of surface roughness on actin filament staining by seeding cells on the Ag–Se coatings with different surface roughness. As shown in Supplementary Fig. S5, the staining of actin filaments significantly increased with surface roughness. However, this was not observed in the Ag and Ag–PTFE coatings. This could be due to their relatively smooth surface that no significant cellular response (e.g. stress) was triggered.

To assess their cytotoxicity, the coated samples were co-incubated with hFOBs and cell viability was determined *via* CVS. Crystal violet binds to proteins and DNA of living cells so that the amount of CVS is directly proportional to the biomass of living cells [50]. Based on the ICP analysis, all the Ag-based coatings released the highest level of Ag^+^ over the first 24 h. As seen in Fig. 5, the presence of Ag-coated sample significantly inhibited cell proliferation by 51.7% when compared with control. The Ag–PTFE and Ag–Se-coated samples demonstrated improved cytocompatibility with cell viabilities of 72.9% and 85.9%, respectively, as compared with control. Interestingly, the Ag–PTFE coating released a slightly lower level of Ag^+^ than the Ag–Se coatings but exhibited higher cytotoxicity against hFOBs. A possible reason is that a residue of fluorocarbon surfactants within the Ag–PTFE coating may release and exert toxic effects on cells. In comparison, the incorporation of fungal-derived SeNPs into Ag coatings led to a slower release of Ag^+^ and improved cytocompatibility.

## Materials and methods

### Materials

*Aureobasidium pullulans* IMI 45533 was obtained from the Geomicrobiology Group, University of Dundee, UK. The 316 L stainless steel plates (10 mm × 10 mm × 1 mm) were purchased from RS Component Ltd (Corby, UK). LIVE/DEAD Baclight bacterial viability kit L13152 was obtained from Fisher Scientific (Loughborough, UK). hFOBs (1.19) were obtained from ATCC (Virginia, USA). All the other materials were obtained from Merck Life Science UK Ltd (Dorset, UK) and used without further purification.

### Fungal formation of Se nanoparticles

*Aureobasidium* *pullulans* were incubated in 100 ml AP1 medium on an orbital shaking incubator at 125 rpm, 25°C in the dark for 10 days. The AP1 medium was prepared according to previous studies [51]. The culture media was harvested and filtered through a cellulose nitrate filter to obtain a clear fungal supernatant. Then, 50 ml fungal supernatant was mixed with 1 ml 1 M sodium selenite solution in a 50 ml falcon tube and reacted for 72 h on an orbital shaking incubator at 125 rpm at room temperature. Particles present in the fungal supernatant were harvested by centrifugation at 13 k× *g* for 30 min until all the particles in the supernatant were completely collected, fungal supernatant was then discarded. A graded ethanol series [50%, 75% and 100% (v/v)] was used for cleaning the harvested particles, each rinse lasting about 15 min, further purification including rinsing harvested particles with 20%(w/v) sodium dodecyl sulphate solution for three times, and finally rinsed three times with sterile Milli-Q water.

### Synthesis of Ag-based nanocomposite coatings

The 316 L stainless steel discs underwent a series of pretreatments according to our previous studies [52] before electroless Ag plating. The method of preparing Ag and Ag–PTFE coatings was previously reported [53] and the concentrations of FC4 and PTFE emulsion in the Ag–PTFE coating bath were 0.5 g/l and 15 ml/l, respectively. To prepare Ag–Se coatings, the above obtained SeNPs suspensions were sonicated at 37 kHz for 10 min and added directly into the coating bath at concentrations of 50 and 100 ml/l, respectively. The plating solution was stirred for 1 min to ensure uniform dispersion of the SeNPs. The coating process was conducted in the dark at room temperature and the coating time was controlled at 5, 10, 20, 30, 40 and 60 min, respectively.

### Characterization

The morphology of harvested SeNPs was characterized using scanning electron microscopy (FE-SEM, JEOL JSM-7400F, Tokyo, Japan) and transmission electron microscopy (TEM, JEOL-1400 plus, JEOL USA, Inc., Peabody, USA). The chemical composition of SeNPs particles was analysed by EDX (QX200, Bruker, USA) and XRD (D2 PHASER, Bruker, USA). The particle size of SeNPs was measured using Image J (LOCI, University of Wisconsin, Wisconsin, USA). The surface morphologies and chemistry compositions of Ag, Ag–PTFE and Ag–Se coatings were characterized by using FE-SEM and EDX, respectively. The distributions of Ag, PTFE and Se were examined by EDX mapping. The Se particle size distribution in coatings was calculated using ImageJ. The coating thickness was measured by using a TESA Micromaster Digital Micrometer (TESA, Renens, Switzerland). Surface wettability of the coatings was determined by measuring static WCAs using a Dataphysics OCA-20 contact angle analyser (DataPhysics Instruments GmbH, Filderstadt, Germany). The release profile of Ag and Se from the coatings was determined by using inductively coupled plasma-optic emission spectrometry (Agilent, California, USA). In brief, the samples (*n* = 3) were separately immersed in 3 ml of serum-supplemented and pure SBF at 37°C for up to 7 days. The media was collected and refreshed every day. The concentrations of Ag and Se in media at 1, 3, 5 and 7 days were measured.

### Corrosion test

Corrosion resistance of all the surfaces was determined *in vitro* by using a CorrTest Electrochemistry Workstation (CS300, Wuhan Corrtest Instrument Co., Ltd, China). The coated samples (working electrodes) were immersed in SBF at 37°C to mimic the biocorrosive environment. Prior to the potentiodynamic polarization test, stable OCPs were obtained for each sample after immersion in SBF for 60 min. Potentiodynamic polarization curves were scanned from −0.5 to 0.5 V with respect to the OCP at a scan rate of 0.5 mV/s. Data fitting and analysis were performed using the software CorrTest^®^ 1.2, corrosion potential (*E*_corr_) and corrosion current density (*i*_corr_) were estimated by Tafel method [54].

### Antibacterial assay

*Staphylococcus* *aureus* F1557 and *E.coli* WT F1693 were used as model bacteria to assess the antibacterial activity of the coatings. Briefly, the inoculum of bacteria was cultured at 37°C in tryptone soya broth (TSB) medium to ∼1 × 10^8^ CFU/ml and diluted to a concentration of ∼2 × 10^6^ CFU/ml in nutrient media [10% TSB in phosphate buffered saline (PBS)]. The samples were sterilized in 70% ethanol and air-dried in dark. After washing extensively with sterile PBS, the samples (*n* = 6) were immersed in 3 ml of the prepared bacterial suspension and incubated at 37°C and 30 rpm for up to 72 h. The nutrient media were collected and refreshed every 24 h and the OD was measured at 600 nm using a spectrophotometer (Biochrom WPA CO8000, Cambridge, UK). After incubation at each time point, the samples were gently rinsed with PBS and transferred into 2.0-ml microfuge tubes with 1.5 ml of PBS and then centrifuged at 2500 rpm for 10 min to detach the bacteria. The bacteria in PBS were then inoculated onto the trypticase soy agar plates and cultured for 20 h before counting. To examine the antibacterial effect, the bacteria attached on surfaces were also stained with SYTO 9 and propidium iodide dyes and observed under a fluorescence microscope (OLYMPUS BX 41, Tokyo, Japan).

### Crystal violet survival assay

All the samples (*n* = 6) were sterilized in 70% ethanol for 2 h and placed in a laminar-flow cabinet to dry. Then, the samples were washed at least three times with sterile PBS and incubated with hFOBs in 48-well plates at a density of ∼1.3 × 104 cells/well. The growth medium for hFOBs consisted of DMEM/F-12, 10% (v/v) FBS, 2.5 mM L-glutamine and 0.3 mg/ml geneticin and the plates were kept in a humidified 5% CO2 atmosphere at 34°C. After 24 h of co-culture, the samples were removed and the media was discarded. The cells in wells were fixed with 1% paraformaldehyde (PFA) for 40 min followed by staining with 0.1% hexamethyl pararosaniline chloride in deionized water at room temperature. After 30 min, the dye was removed, and the wells were thoroughly washed with deionized water and air-dried before the addition of acidified methanol. The absorbance was recorded at 595 nm using a microplate reader. Wells containing only cells were used as control and the data were normalized to the control group using the formula: Cell viability (%) = (absorbance value of sample group/absorbance value of control group) × 100%.

### Cell adhesion

The sterile samples (*n* = 6) were placed in 24-well plates and a 1.0 ml complete medium containing ∼1 × 10^5^ cells was seeded onto each sample. After 72 of incubation, the samples were gently rinsed with PBS and the cells were fixed with 4% PFA for 30 min. The samples were then treated with 0.1% Triton-X 100 for 25 min and stained with Alexa Fluor 488 phalloidin and DAPI in dark at room temperature. The samples were then thoroughly rinsed with PBS and the cell morphology was observed using a confocal laser scanning microscope (TCS SP8, Leica, Wetzlar, Germany).

### Statistical analysis

All data were analysed using one-way ANOVA and represented as means ± standard deviation. *P *<* *0.05 was considered significant and *P *<* *0.01 was considered highly significant.

## Conclusions

In this work, SeNPs were synthesized *via* a fungi-mediated biorecovery approach and doped in Ag coatings on 316 L stainless steel *via* electroless plating process. The SeNPs exhibited superior stability in the plating solution and led to an increase in the Ag particle size, resulting in improved corrosion resistance and a controlled release of Ag^+^. However, it should be noted the stabilizing materials (protein molecules) with the SeNPs were not fully studied in this work and their potential biological response remained to be explored. Despite trace amounts of Se were also released, their antibacterial activity against planktonic *S.aureus* was not detected. The Ag–Se coating was shown to be more effective in inhibiting bacterial growth and bacterial adhesion on surface compared with Ag and Ag–PTFE coatings. After 3 days co-incubation, the Ag–Se coating reduced 81.2% and 59.7% of viable bacterial adhesion when compared with Ag and Ag–PTFE-coated surfaces, respectively. The antibacterial mechanism of Ag–Se coatings remains to be further investigated but the coating displayed an effective contact-killing activity against *S.aureus* in this study. The Ag–Se-coated samples also showed less cytotoxicity than Ag and Ag–PTFE coatings, indicating their potential to become a new type of anti-infection coating to prevent PTIs.

## Supplementary data

Supplementary data are available at *REGBIO* online.

## Funding

This work was supported by the UK Engineering and Physical Sciences Research Council (EP/P00301X/1); the European Union’s INTERREG VA Programme (the Bryden Centre project, Project ID VA5048), which was managed by the Special EU Programmes Body (SEUPB), with match funding provided by the Department for the Economy in Northern Ireland and the Department of Business, Enterprise and Innovation in the Republic of Ireland.

*Conflict of interest statement*. None declared.

## Supplementary Material

rbac013_Supplementary_Data
