# Supplementary material for: Fungal-derived selenium nanoparticles and their potential applications in electroless silver coatings for preventing pin-tract infections
Source: Regen Biomater. 2022 Feb 22;9:rbac013. doi: 10.1093/rb/rbac013 (PMC9017370; doi:10.1093/rb/rbac013)
Supplement: rbac013_Supplementary_Data [file rbac013_supplementary_data.docx]

Fungal-derived Selenium Nanoparticles and Their Potential Applications in Electroless Silver Coatings for Preventing Pin-tract Infections

Xinjin Liang ^1, 2^, Shuai Zhang ^3,^ *, Geoffrey Michael Gadd ^2, 4^, John McGrath ^5^, David W Rooney ^6^, Qi Zhao ^7,^*

^1^The Bryden Centre, School of Chemical and Chemistry Engineering, Queen’s University Belfast, Belfast, BT7 1NN, UK

^2^Geomicrobiology Group, School of Life Sciences, University of Dundee, Dundee, DD1 5EH, UK

^3^School of Pharmacy, Queen’s University Belfast, BT9 7BL, Belfast, UK

^4^State Key Laboratory of Heavy Oil Processing, Beijing Key Laboratory of Oil and Gas Pollution Control, College of Chemical Engineering and Environment, China University of Petroleum, 18 Fuxue Road, Changping District, Beijing 102249, China

^5^School of Biological Sciences, Queen's University Belfast, Belfast, BT9 5DL, United Kingdom

^6^ School of Chemistry and Chemical Engineering, Queen’s University Belfast, Belfast, BT9 5AG, Northern Ireland, UK

^7^ School of Science and Engineering, University of Dundee, Dundee, DD1 4HN, UK

*** Corresponding Authors**:

Email addresses: [shuai.zhang@qub.ac.uk](mailto:shuai.zhang@qub.ac.uk) (S Zhang).

[q.zhao@dundee.ac.uk](mailto:q.zhao@dundee.ac.uk) (Q Zhao)


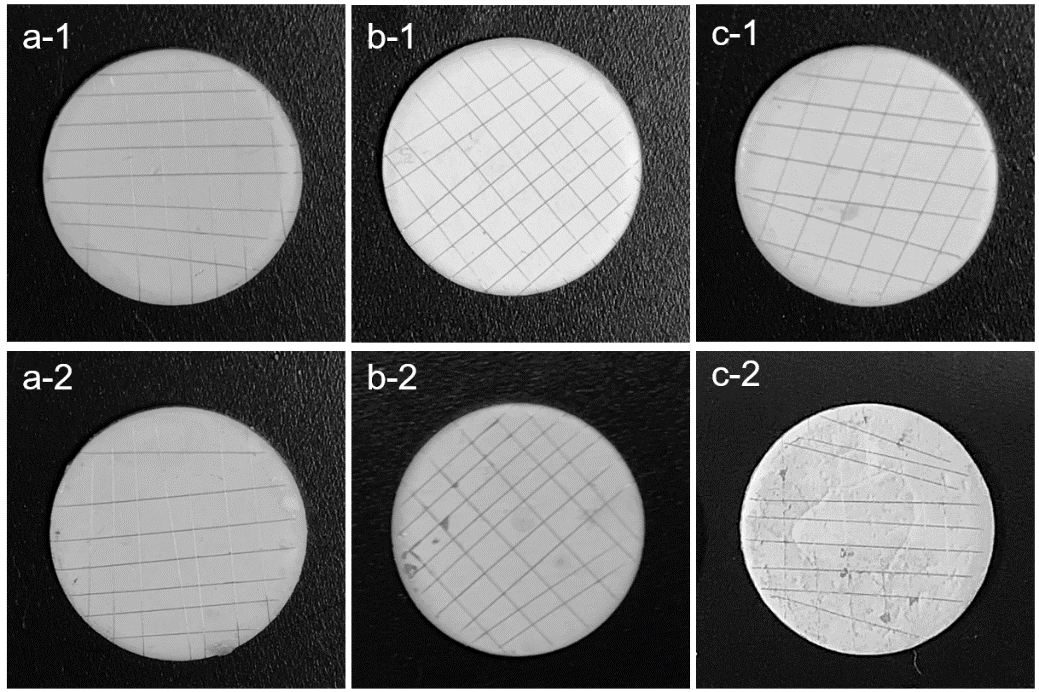


Figure S1. Tape test results for different coatings. (a-1) Ag coating, (b-1) Ag-PTFE coating and (c-1) Ag-Se (10ml/L) coating with a deposition time of 1. 5 h; (a-2) Ag coating, (b-2) Ag-PTFE coating and (c-2) Ag-Se (10ml/L) coating with a deposition time of 2 h.


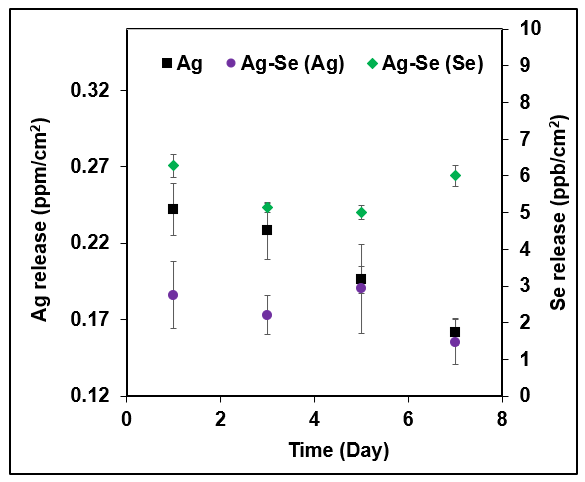


Figure S2. Ag and Se release profiles in the serum-supplemented SBF over time.

Table S1. Surface roughness of Ag and Ag-Se (10ml/L) coatings with deposition time

|  | Deposition time (h) | | |
| --- | --- | --- | --- |
|  | 0.5 | 1 | 1.5 |
| Ag | 0.17 | 0.43 | 0.92 |
| Ag-PTFE | 0.25 | 0.69 | 1.06 |
| Ag-Se | 0.49 | 1.17 | 3.31 |


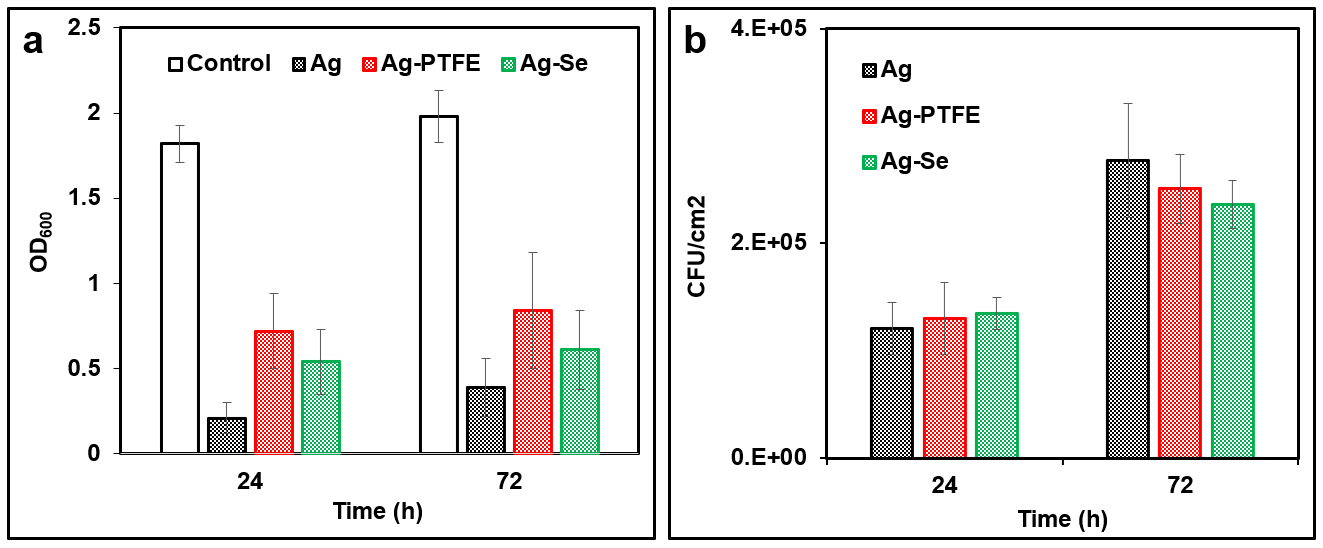


Figure S3. (a) *E. coli* growth in the presence of Ag, Ag-PTFE and Ag-Se (100ml/L) coated samples after 24 h and 72 h incubation; (b) Numbers of adhered *E. coli* on different surfaces after 24 h and 72 h incubation.

**
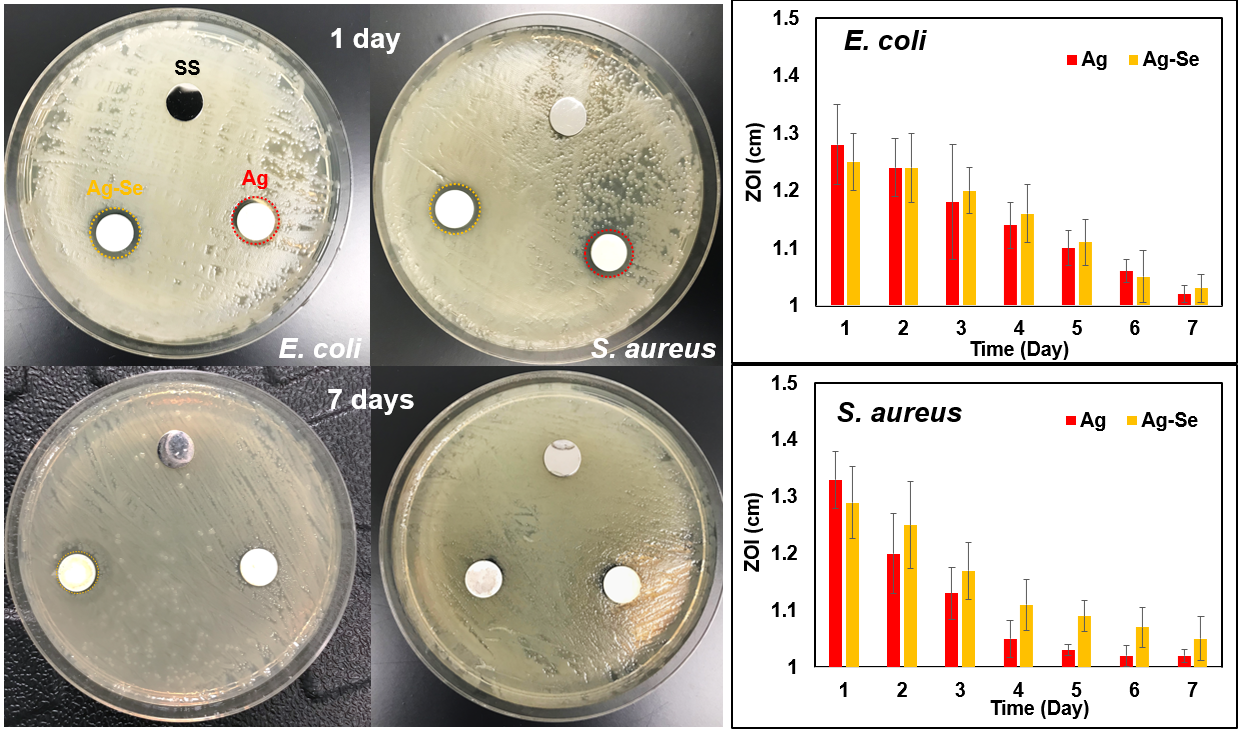
**

Figure S4. Zone of inhibition (ZOI) test against *E. coli* and *S. aureus* and diameters of ZOI.


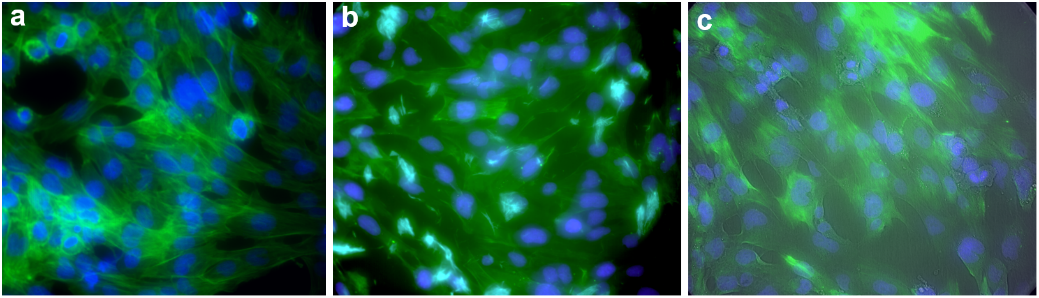


Figure S5. Typical confocal microscopy images of hFobs attached on Ag-Se surfaces with Ra val of (a) 0.49 μm (b) 1.17 μm and (c) 3.31 μm after 36 h incubation.
